# Evaluation of Safety and Efficacy of Ferric Carboxymaltose for the Treatment of Iron Deficiency Anaemia in Pregnancy and Postpartum: A Retrospective Observational Study

**DOI:** 10.7759/cureus.100691

**Published:** 2026-01-03

**Authors:** Pooja Yadav, Saloni Kamboj, K Aparna Sharma, Anubhuti Rana, Neena Malhotra

**Affiliations:** 1 Obstetrics and Gynaecology, All India Institute of Medical Sciences, New Delhi, New Delhi, IND

**Keywords:** efficacy, ferric carboxymaltose, iron deficiency anemia, postpartum, pregnancy, safety

## Abstract

Objectives

The objective of this study is to evaluate the safety and efficacy of ferric carboxymaltose (FCM) in the treatment of iron deficiency anaemia in pregnant and postpartum women.

Materials and methods

A retrospective observational study conducted from March 2023 to March 2024 at a tertiary care hospital, where 153 pregnant and postpartum women with iron deficiency anaemia (Hb 6 g/dL to 10.9 g/dL) were recruited. The participants received a calculated dose of injection FCM. The primary outcome was a rise in haemoglobin from baseline at two weeks, four weeks, six weeks, and 12 weeks. Secondary outcomes were safety, adverse events, and adverse perinatal outcomes following the injection of FCM.

Results

The mean haemoglobin rise was 1.06 ± 0.50 g/dL, 1.80 ± 0.62 g/dL, 2.24 ± 0.94 g/dL, and 3.23 ± 1.21 g/dL at two weeks, four weeks, six weeks, and >6 weeks, respectively (p value < 0.001). Around 52% participants became non-anaemic, and 33% improved to mild anaemia from moderate anaemia post-FCM therapy. Among pregnant women with severe anaemia, a rise in haemoglobin from 2 g/dL to 4.7 g/dL was noted at six to eight weeks of follow-up. No major adverse events were noted.

Conclusion

Intravenous FCM is a safe and effective treatment option for iron deficiency anaemia in pregnant and postpartum women, with a single infusion required, with no serious adverse events, improving the overall patient compliance.

## Introduction

Anaemia is one of the major health concerns all over the world, with iron deficiency being the most common cause of anaemia. The global prevalence of anaemia in the reproductive age group is 29.6% whereas it is 36.5% in pregnancy [1]. According to the National Family Health Survey-5, around 52% pregnant women in India are anaemic [2]. Iron deficiency anaemia not only adversely affects maternal health but is also associated with preterm delivery, low birth weight infants and low iron stores in neonates, leading to impaired development [1,3]. About half of maternal mortality in South Asian countries is due to anaemia, and India contributes to 80% of the entire volume [4]. Despite upgrading treatment strategies regularly, the prevalence of anaemia has been almost stagnant for the last two decades [2,5]. Oral iron therapy is recommended for prophylaxis and treatment of mild anaemia during pregnancy, but efficacy is compromised by lack of absorption and poor compliance due to gastrointestinal side effects. Therefore, oral iron therapy is not a sufficient treatment, especially for moderate and severe anaemia. Also, blood transfusion has its own challenges of adverse events, limited availability and risk of transmission of blood-borne infections. Parenteral iron therapy has been shown to provide a better response in women in antenatal and postpartum, where prompt correction of anaemia is required and decreases the need for blood transfusion [6-7]. Intravenous iron sucrose has been in use for a long time, with negligible safety issues. The only disadvantage, however, is the maximum dose per sitting, which is 300 mg per sitting or 600 mg per week. This necessitates multiple hospital visits, adding to the cost of therapy. Various studies have been published on relatively recent parenteral iron, ferric carboxymaltose (FCM), in the treatment of anaemia in the reproductive age group, including postpartum women, but there is a paucity of studies on safety and efficacy during pregnancy. The present study aims to evaluate the safety and efficacy of FCM in moderate to severe anaemia in pregnant and postpartum women.

## Materials and methods

The retrospective observational study was conducted in the Department of Obstetrics and Gynaecology of All India Institute of Medical Sciences (AIIMS), New Delhi, from March 2023 to March 2024. The Institutional Ethics Committee provided the clearance for the study. Pregnant women in the second and third trimester with a gestation period of less than 37 weeks and postpartum women who delivered at the institute with haemoglobin levels between 6 g/dL and 10.9 g/dL were included in the study. The exclusion criteria were anaemia other than iron deficiency anaemia, chronic infection including Hepatitis B and Hepatitis C, chronic liver disease, chronic kidney disease, and a history of allergic reaction with parenteral iron.

On recruitment, a record review was performed to note the detailed clinical history and general, systemic and obstetric examination. The baseline haematological investigations were noted including complete blood count, peripheral smear, mean corpuscular volume (MCV), mean corpuscular haemoglobin (MCH), mean corpuscular haemoglobin concentration (MCHC), serum iron, serum ferritin, serum total iron binding capacity transferrin saturation and high-performance liquid chromatography (HPLC) and also other baseline investigations were kidney function test and liver function test.

Iron requirements were calculated using Ganzoni’s formula:

Iron deficit (mg) = 0.24 × pre-pregnancy weight (kg) × (target Hb - actual Hb) (g/dL) + storage iron (1000 mg) for a target Hb of 14 g/dL

Storage iron was taken as 1000 mg instead of 500 mg in Ganzoni’s formula.

After calculating the total iron deficit, participants were administered intravenous FCM as an infusion. The maximum dose per sitting was 1000 mg, which was diluted in 100 mL of 0.9% normal saline and administered as an intravenous infusion over 15 minutes. Subsequent doses, if needed, were planned on day 7 and day 14. The general condition of the patient, pulse, temperature, oxygen saturation and blood pressure were noted before infusion, at five minutes and after the infusion. Foetal heart rate monitoring was done before and after infusion. Any minor or major adverse effects were noted. The haemoglobin values were recorded at two weeks, four weeks, six weeks, and 12 weeks post-infusion with haemoglobin. The primary outcome was the change in haemoglobin from baseline at two weeks, four weeks, six weeks, and 12 weeks. Secondary outcomes were safety, adverse effects and adverse perinatal outcomes following FCM infusion.

Data were expressed as mean ± SD, number of patients (percentage), or median (range), as appropriate. Continuous variables were analysed using a Student's t-test for independent samples. Results were considered to be statistically significant when the p-value < 0.05.

## Results

A total of 153 women (135 pregnant and 18 postpartum women) meeting the inclusion and exclusion criteria were included. The mean age of participants was 32.6 years (range 20 to 43 years), the mean haemoglobin was 8.82 g/dL (range 6.2 to 10.9 g/dL) (Table 1), the percentage of women with mild, moderate and severe anaemia was 16% (N = 25), 80% (N = 122), and 4% (N = 6), respectively.

**Table 1:** Baseline haemoglobin and mean POG at FCM infusion FCM, ferric carboxymaltose; POG, period of gestation

Parameters	N	Mean ± SD	Median (QR)	Range
Period of gestation (weeks)	135	32.66 ± 2.73	33 (31,34)	23-37
Baseline haemoglobin (g/dL)	135	8.82 ± 0.96	8.8 (8.2,9.5)	6.2-10.9

**Figure 1:**
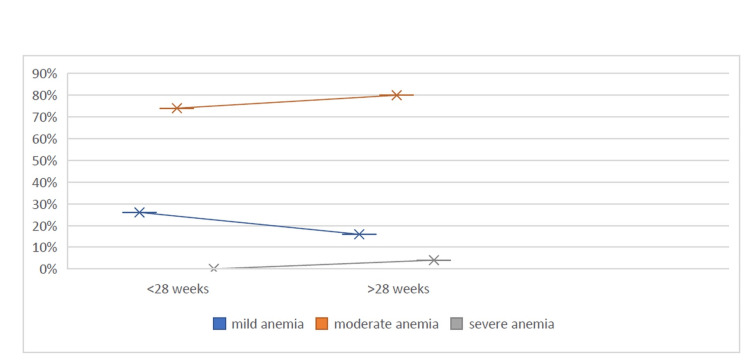
Trend of severity of anaemia with advancing gestation

As shown in Figure 1, among pregnant women, prevalence of mild and moderate anaemia was 26% (N = 35) and 74% (N = 100), respectively, and no women had severe anaemia in women with <28 weeks gestation, whereas it was 16% (N = 22), 80% (N = 108) and 4% (N = 5), respectively, in women with >28 weeks gestation.

The mean serum ferritin of participants was 11.9 ng/mL (1.6 ng/mL to 25 ng/mL). Around 75% (N = 115) of participants had serum ferritin < 15 ng/mL, and 23.5% (N = 36) had serum ferritin < 30 ng/mL. The mean period of gestation (POG) at FCM infusion was 32.6 weeks (range 23 to 37 weeks) and postpartum day 6 (day 4 to day 9) among antenatal and postpartum women, respectively. The mean dose of FCM was 1000 mg; two participants required a second dose of FCM, 500 mg given after one week. The mean duration to complete the treatment was one week, and the mean number of visits required was one.

The baseline haemoglobin and rise in haemoglobin at two weeks, four weeks, six weeks, and >6 weeks, as shown in Table 2 (using paired t-test).

**Table 2:** Rise in Hb at follow-up visits after FCM infusion FCM, ferric carboxymaltose

Follow-up interval	Number of patients (N)	Baseline Hb (g/dL) mean ± SD	Follow-up Hb (g/dL) mean ± SD	Mean rise in Hb (g/dL) ± SD	t-value (df)	p-value
≤2 weeks	23	9.23 ± 0.71	10.29 ± 0.80	1.06 ± 0.50	10.17 (22)	<0.001
>2 to ≤4 weeks	50	8.92 ± 0.91	10.72 ± 0.84	1.80 ± 0.62	20.53 (49)	<0.001
>4 to ≤6 weeks	51	8.82 ± 1.01	11.06 ± 0.77	2.24 ± 0.94	17.02 (50)	<0.001
>6 weeks	29	8.41 ± 0.99	11.64 ± 0.89	3.23 ± 1.21	14.38 (28)	<0.001

The patients followed up according to their scheduled antenatal visit, emergency visit to the labour ward, or postnatal follow-up for maternal or newborn concerns, and their haemoglobin was noted. The haemoglobin mean rise was (1.06 ± 0.50) g/dL, (1.80 ± 0.62) g/dL, (2.24 ± 0.94) g/dL, (3.23 ± 1.21) g/dL at two weeks, four weeks, six weeks and >6 weeks, respectively (p < 0.001). The target haemoglobin after FCM infusion was taken as 11g/dL. About 52% (N = 80) of participants achieved the target Hb of 11 g/dL or more by 12 weeks of FCM infusion (among these 80 participants, 51 participants achieved the target Hb by six weeks, and a further 29 participants achieved it by 12 weeks). Around 1.3% (N = 2) of participants with severe anaemia and 33% (N = 51) with moderate anaemia became mild anaemia post-therapy. The mean duration required to increase Hb by 2 g/dL was 5.2 ± 2.6 weeks.

Five pregnant women with severe anaemia received FCM, and a rise in Hb is shown in Table 3.

**Table 3:** Period of gestation, baseline Hb, and rise in Hb in women with severe anaemia FCM, ferric carboxymaltose; POG, period of gestation

POG at FCM infusion (weeks)	Baseline Hb (g/dL)	Follow-up (weeks)	Rise in Hb (g/dL)
31 weeks	7.0 g/dL	8 weeks	2.0 g/dL
30 weeks	6.4 g/dL	6 weeks	4.0 g/dL
32 weeks	7.0 g/dL	8 weeks	4.7 g/dL
34 weeks	6.5 g/dL	6 weeks	3.7 g/dL
33 weeks	6.2 g/dL	6 weeks	3.2 g/dL

Beta thalassemia trait was found in two participants. There were no serious maternal foetal adverse events. Approximately 90% (N = 122) of the participants had neonates appropriate for gestational age, 9% (N = 12) of the neonates were small for gestational age, as all these had foetal growth restriction during pregnancy, and one GDM participant delivered a large for gestational age newborn. Only one participant had a preterm vaginal delivery (eight weeks after FCM infusion).

## Discussion

The present study was conducted to see the safety and efficacy of intravenous FCM during pregnancy and the postpartum period in women with moderate to severe anaemia. Iron sucrose has been in practice in the management of anaemia in pregnancy as a part of standard care with negligible side effects, but the only concern is the limited dose per sitting, requiring multiple hospital visits. FCM, on the other hand, can be given in a larger dose in a single sitting. In our study, FCM gave promising results in view of safety and tolerability during pregnancy and postpartum, validated by various global [8-10] and Indian studies [6,11-13]. In the present study, FCM was given under residents’ supervision in hospital wards with emergency care available. None of the patients required emergency care. FCM is a non-dextran iron molecule having very low immunogenic potential [14]. The structure of the macromolecule favours its guarded release into the reticuloendothelial system with negligible risk of a large rush of ionic iron into the blood [15]. Although FCM does not cross the placenta [16], as a measure of precaution, parenteral iron, including FCM, is contraindicated during the first trimester.

The mean rise in haemoglobin at two weeks, four weeks, six weeks, and >6 weeks follow-up was 1.06 g/dL, 1.8 g/dL, 2.2 g/dL, and 3.2 g/dL, respectively, which was statistically significant.

Similar to our study, the study by Jose et al. [6] showed a mean rise of Hb by 2.9 g/dL by 12 weeks at baseline Hb of 8.5 g/dL. In the present study, the rise of haemoglobin ranged from 2 g/dL to 4.7 g/dL in severe anaemia at six to eight weeks after FCM. Breymann et al.’s study showed a 3.3 g/dL rise in mean haemoglobin at 12 weeks, which is consistent with our study [9].

Our study showed that 80% of included women had moderate anaemia 4% had severe inferring moderate to severe anaemia is a significant health concern in India and increase in women with moderate and severe anaemia in third trimester as compared to second trimester due to increase in iron demand as majority of iron transfer to foetus occurs during this time indicates the importance of timely diagnosis and management of mild to moderate anaemia. Body iron stores are largely determined by the serum ferritin levels. The majority of participants had serum ferritin levels below 15 ng/mL, comparable to other Indian studies [6,14,17-19] but lower than the study by Breymann et al. [9], and Van Wyck et al. [19], possibly due to dietary iron deficiency, delay in seeking healthcare services and social status as compared to high-income countries. Considering the poor body iron stores in our population, we took a higher value of target haemoglobin (14 g/dL instead of 12 g/dL) and added 1000 mg to iron stores instead of 500 mg. The mean POG of women receiving FCM in the present study was 32.6 weeks, the mean duration to complete the treatment was one week, and the number of visits required was one. This is of particular help in resource-limited countries like India, where women present for their first antenatal visit in late gestation and parenteral iron preparation FCM, having qualities of a good safety window, rapid action, long-term effect, and larger dose in a single sitting, becomes a good therapeutic option for correction of iron deficiency anaemia in the third trimester. In the current study, 16% of participants had mild anaemia, and parenteral iron therapy was considered due to intolerance to oral iron therapy with low serum ferritin levels. The drug in the present study was available free of cost under the Janani Shishu Suraksha Karyakaram (JSSK) programme by the Government of India, but considering patients’ travel cost and lost wages due to absence from work and healthcare burden in repeated interventions, it should be cost-effective.

The study was limited by being a single-centre study, a retrospective design, and limited participants; however, it provides scope for multicentric randomised studies with a larger sample size.

## Conclusions

Intravenous FCM is safe, well-tolerated, and effective in treating iron deficiency anaemia in pregnancy with no serious adverse maternal or foetal outcomes. It has the advantage of a larger dose per sitting, hence a lesser dose and fewer hospital visits required. Significantly shorter duration of treatment makes the drug suitable for use in anaemia in the third trimester, as well as results in improved patient compliance.
